# Dr. Stephen Fullom Conolly

**Published:** 1911-09-30

**Authors:** 


The death is announced of Dr. Stephen Fullom Conolly, of North Kensington, in his sixty-second year. Dr. Conolly was an old student of Charing Cross Hospital, and became a member of the Royal College of Surgeons in 1871.

